# Photosensitivity of Dispersing Cryptic Date Stone Beetles *Coccotrypes dactyliperda* (Coleoptera, Curculionidae, Scolytinae)—A Pilot Study

**DOI:** 10.3390/insects13090851

**Published:** 2022-09-19

**Authors:** Dirk H. R. Spennemann

**Affiliations:** School of Agricultural, Environmental and Veterinary Sciences, Charles Sturt University, P.O. Box 789, Albury, NSW 2640, Australia; dspennemann@csu.edu.au

**Keywords:** scolytid beetles, photophobia, cryptic species, color vision, crawl speeds

## Abstract

**Simple Summary:**

Date stone beetles (*Coccotrypes dactyliperda*), which feed on the albumen inside palm seeds, are a major pest in date palm plantations in the Middle East and North Africa. While they spend most of their life cycle in darkness inside the brood galleries where they mate and reproduce, females emerge for a short period of time when the brood gallery has become too crowded or when the seed’s albumen has been exhausted. During this emergence they seek out fresh seeds, commonly by attacking unripe dates still on the palm. Previous work indicated that the beetles might prefer dark conditions (i.e., nighttime) when emerging from seeds, whereas anecdotal evidence suggested that the beetles might be attracted to light. The controlled experiments showed that the beetles, once in the open, are attracted by and move faster to a light source, but that the color of the surface (red, blue, green, black) has no influence on either direction or crawl speed.

**Abstract:**

The date stone beetle, *Coccotrypes dactyliperda*, is a cryptic spermatophagus species that spends almost its entire life cycle inside the seeds of palms, esp. *Phoenix* sp. Only during dispersal, when the host seed has been largely eaten out, do females emerge for a short period of time in search of a fresh seed in which to establish new brood galleries. Previous work indicated that *C. dactyliperda* might be photophobic, preferring to emerge from seeds during night hours, whereas anecdotal evidence suggested that the beetles might be photophilic in terms of their movements post emergence. This paper examines the photosensitivity of the species under controlled laboratory conditions. The results show that *C. dactyliperda*, once removed from the brood chamber, is attracted by and moves faster to a light source, but that the color of the lit surface (red, blue, green, black) has no influence on either direction or crawl speed.

## 1. Introduction

The date stone beetle, *Coccotrypes dactyliperda* (Fabricius, 1801), is a cryptic spermatophagus beetle (Coleoptera: Curculionidae: Scolytinae: Dryocoetini), with females measuring 1.9 to 2.2 mm in length and about 0.7 to 1 mm in width. The beetle, which has a convex appearance and is hairy across the dorsal surface, ranges in color from reddish brown to almost black-brown [1,2,3,4]. When in their breeding galleries, *C. dactyliperda* have been shown to be quite resilient to exposure to pesticides [5], weak acids [6] and short-term exposure to subzero temperatures [7].

Initially distributed in the Middle East and North Africa as part of the date palm horticultural complex, the distribution range of the species has seen a remarkable increase during the nineteenth century, mainly due to the trade in date palms (*Phoenix dactylifera* L., 1783 (Arecales: Arecaceae)) as fruit for human consumption, the distribution of palm seeds (in particular *P. canariensis*) (Chabaud, 1882) (Arecales: Arecaceae) for horticultural endeavors and in the form of vegetable ivory for button manufacture. Today, *C. dactyliperda* has become a true cosmopolitan species that can be found in most subtropical and temperate zones [8].

The beetle attacks the green drupes (fruit) of the date palm (*P. dactylifera*), causing the bulk of these to abscise (naturally fall off) one to two days later [9] causing production losses between 20 and 40% [10,11]. *C. dactyliperda* also attack other palms, in particular the Canary Island date palm (*P. canariensis*), the doum Palm (*Hyphaene thebaica* L.) and tagua palms (*Phytelephas* sp.) [12].

*Coccotrypes dactyliperda* spends almost its entire life cycle inside the seeds of palms [13]. Only during dispersal do females emerge for a short period of time, seeking out new seeds. In experimental settings, this exposure to the open lasts between 4 and 48 h [14]. The beetles tend to infest both drupes on the tree and seeds fallen to the ground, as well as seeds of fallen dates, often after the pericarp has been consumed by other animals, such as rodents. El-Sufty and Helal [15] assert that the beetles prefer to crawl up the stem of the palm and then along the inflorescence to reach the drupes, rather than to fly. *C. dactyliperda* is not a ready flyer but takes to flight when either crowded or disturbed.

While a horizontal dispersal capacity of the genus of ~50 m, presumably by flight, has been documented under field conditions [16] and dispersal over 350 m has been recorded [17], their flight is usually limited to short distance movements of 150 mm or less, primarily in crowded conditions [12,15]. Consequently, the majority of dispersal movements occurs between nearby seeds, followed by palms in close proximity with lateral movements usually less than 4–5 m [17].

Given that the behavior of *C. dactyliperda* is primarily cryptic, it is of little surprise that there are only very few published observations that examine the behavior of the species. The tunnelling behavior was examined by Adolf Herfs [18,19], who primarily commented on factors such as hardness of seeds and traction, and by Spennemann [20,21], who examined aspects of gnawing action and traction. Other experiments forced, or observed, *C. dactyliperda* to crawl from the centers of circular filter papers but did not consider or record influences of light on the direction of movement [5,22].

To date, no work has been carried out on the physiology of the visual system of *C. dactyliperda* or its overall vision capabilities. The only work which in the widest sense refers to this is that of Bright [23], who reported on a cavernicolous population of *C. dactyliperda* in Trinidad. Here, the species underwent an adaptation to the dark cave environment by losing both the definition of the eye as well as a number of facets. 

The literature on *C. dactyliperda* universally comments on kairomones from date seeds, especially the alcohol mediated fractions, as cues when searching for new host locations [24,25]. While the primary cues for host selection by scolytid beetles are olfactory, some scolytid species, in particular wood borers (*Xyleborus* sp.), also use visual cues during dispersal, such as silhouette and stem diameter [26,27,28]. Like other insects, scolytid beetles have shown photosensitivity to different wavelengths [28,29], in particular blue (400–470 nm) and green light (500–530 nm) [30,31]. Other studies of scolytids, in particular bark beetles, have found that red [32] and in particular black trap colors were more attractive [33,34,35]. Several studies suggest it is less the hue of the color but the reflectance that acts as an attractant, with low reflectance colors preferred [27,36,37,38,39]. Color preferences are not universal among bark beetles, however. Rather, they are species-dependent and likely to be correlated with host patterns. Green traps (with a higher level of reflectance), for example, were favored by *Hypocryphalus mangifera* (Stebbing) infesting Mango trees [40].

While these studies have informative value, they cannot be readily ported to *C. dactyliperda*. The bulk of the cited color studies relies on airborne specimens, commonly trapped in colored pheromone traps placed between 0.5 and 1.7 m above ground. As noted, *C. dactyliperda*, however, primarily disperses by crawling and tends to be a reluctant flier.

In a food preference and breeding experiment, Spennemann [14] observed that emergence occurred statistically significantly more frequently during the hours of the night than of the day. As there were no external factors (no differences in temperature or humidity) between artificial day and night, the author speculated that the lack of light at night initiated and facilitated the emergence and that this preference may have been an instinctive predator avoidance strategy as all known predators are diurnal birds [12].

During laboratory work in preparation of various experiments [6,7,21] it was noted, however, that *C. dactyliperda* being counted out from a tilted white ceramic bowl consistently climbed up the light side, while avoiding the shadier side. A similar movement towards the lighter side was observed in beetles placed in translucent food storage containers. It was therefore decided to formally test whether *C. dactyliperda* would be attracted to light sources. This paper will describe three experiments to investigate the photosensitivity of *C. dactyliperda* in ambient light and in darkness with a point light source. 

## 2. Methodology

### 2.1. Origin of Samples

The *C. dactyliperda* specimens used for the follow-up experiment were derived from the same population as those used for the 2018 food preference and resilience experiments [6,7,14]. These beetles had infested (undetected) one or more sample seeds set aside in storage for future germination and had multiplied in these seeds between December 2018 and February 2020. That population was then used both for experimentation and for further breeding. The beetles, all females, used for the experiments were collected from the breeding containers after they had (voluntarily) emerged from the seeds in which they had bred. Thus, they can be regarded as representative of beetles searching for new seeds to colonize. The collected beetles were aggregated into vials of 100 individuals each. The vials were filled in batches of five, with beetles added at random until each vial had the same complement of beetles.

### 2.2. Set-Up

Given the lockdown and movement restrictions due to COVID-19 in April 2020, the experiment was carried out in an ad hoc laboratory setting in a non-residential basement at the author’s residence. The experiments were carried out in the middle of the day, with ambient room temperatures between 20 °C and 23 °C. Indirect light (overcast skies) entered the semi-darkened ad hoc laboratory through a small window (for experiment #1).

#### 2.2.1. Experiment #1

**Figure 1:**
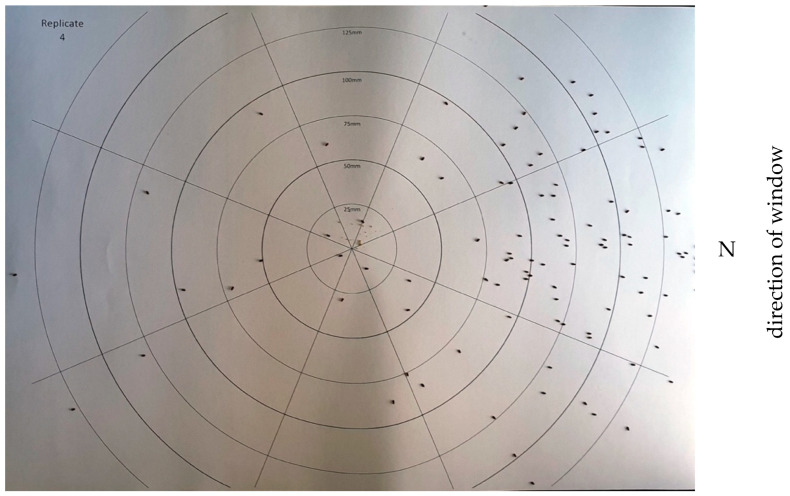
Example of a recording aid (experiment #1, replicate #4 in progress). Note the differences in light on the surface. Octant N is at the right.

A single experimental chamber was constructed from a 35 L clear PVC storage container (520 × 360 × 200 mm) where three internal sides had been sprayed with matt black paint. The fourth (narrow) side was left untreated, allowing diffused light to penetrate into the chamber. The chamber was set up on a workbench in a semi-darkened room with the untreated side of the chamber facing the small window (9.2 ± 1.7 lx). As there was no lid, low-level ambient diffused light (5.8 lx) could enter the experimental chamber from the top. A piece of A3-sized white paper printed with a recording aid was placed into the center. The recording aid comprised of concentric circles (at 25 mm intervals) divided into octants (Figure 1). At the start of each experiment, 100 *C. dactyliperda* individuals were released into the center of the circles (tipped out of a vial without time to acclimatize to the light conditions) and left to walk at their own pace and direction. Each replicate was terminated after 3 min, at which time the beetles had dispersed. Each cohort of 100 beetles was only used once and euthanized at the termination of the experiment to ensure that the replicates were independent. To prevent olfactory stimuli of beetles crawling across the recording aid from influencing subsequent replicates, a clean recording aid was used for each replicate. In total, ten replicates were carried out over two days, with five replicates each at the same time of day (between 13:30 h and 13:50 h).

#### 2.2.2. Experiment #2

The experimental chamber used for experiment #1 was modified by also blacking out the fourth side. Colored 120 gsm cardboard (A4 sheets folded sideways) was placed in three of the sectors. Each color covered half of a long and half of a short side of the chamber from the base of the side to the top. The fourth sector remained matte black (as control). The colors were red (NCS 1070-R10B, luminosity 8.3 ± 0.5 lx), blue (NCS 3060-R70B, 5.8 ± 0.3 lx) and green (NCS 2060-G, 8.9 ± 0.3 lx) [41]. The placement order of the colors was randomized. Each color was assigned a number between 1 and 4 and the sequence determined by the first occurrence in a sequence of ten numbers generated with the RANDBETWEEN function in MS Excel (see Table 1).

As with experiment #1, a piece of A3-sized white paper printed with a recording aid was placed into the center. The experimental chamber was illuminated with low-level, diffused ambient light from the open top (the same conditions as experiment #1). Ten replicates were carried out, again with 100 *C. dactyliperda* per replicate. Each cohort of 100 beetles was only used once. At the end of each replicate the cohort of beetles was retained for experiment #3. As with experiment #1, a clean recording aid was used for each replicate.

#### 2.2.3. Experiment #3

**Figure 2:**
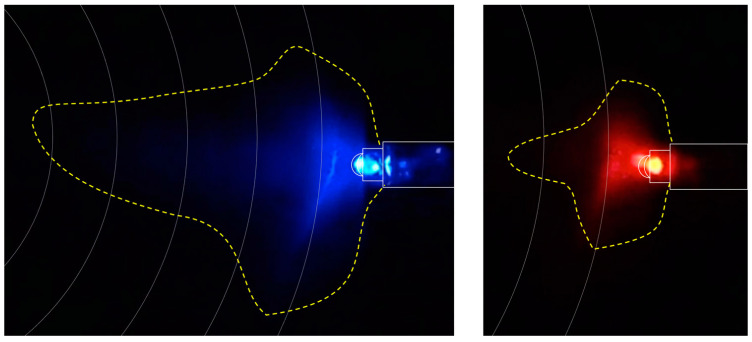
Light cones emitted by the diodes. Section of screenshots of videos taken during experiment #3 (mono color experiments). The dotted line indicates the range of the light cone as determined via an extreme enhancement of shadows in Photoshop. The concentric circles indicate distances (in 25 mm intervals) from the center (compare Figure 4).

**Figure 3:**
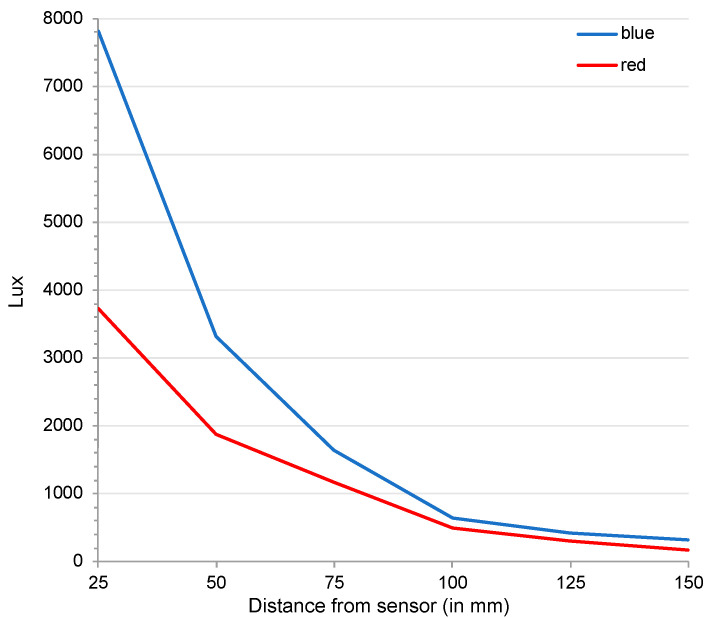
Attenuation of the luminosity of red and blue LEDs. Value for 0 mm not plotted (both 103,520 lx).

**Figure 4:**
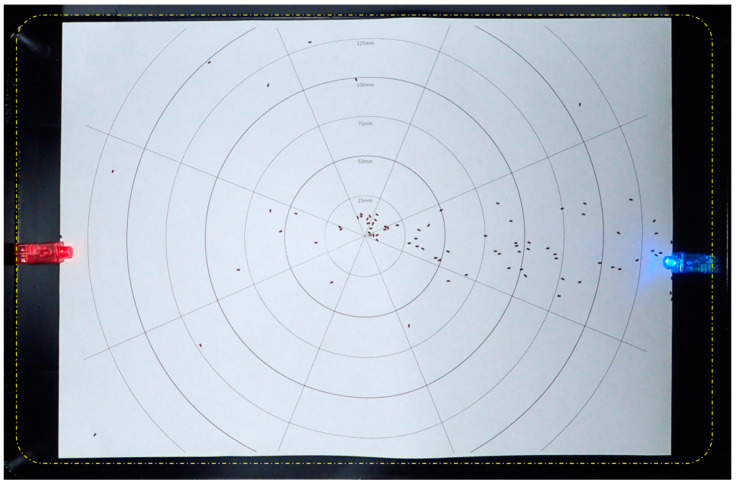
Example of recording (experiment #3, bi-color, replicate #3 in progress). Flash image taken 104 s after start. The dashed yellow line demarcates the flat base of the experiment container. Octant N is at the right.

The experimental chamber and the recording aids were the same as those used for experiment #2. Colored LED point sources (red 620–630 nm and blue 460–470 nm) with a light beam diffused and reflected on the white paper surface (Figure 2 and Figure 3) were inserted at the 200 mm circle of the right octant (coded as N) (red; blue, three replicates each) and at both right (coded as N, blue light) and left octants (coded as S, red light) (three replicates) (Figure 4). The experiment was carried out in full darkness, at night with the room without lights. Position recording occurred via video (replicate #1 for each sub-experiment) with flash photography at irregular, approximately 25–30 s intervals. As with experiment #1, a clean recording aid was used for each replicate. At the termination of the experiment, each cohort of beetles, which had been previously used in experiment #2, was euthanized. As the experiments were conducted in total darkness, crawl speeds could not be recorded.

### 2.3. Recording

The experiments were recorded (both still photos and video) with an Olympus TG-3 camera with a Sony 16-megapixel, 1/2.3 inch backside-illuminated CMOS sensor, which was tripod mounted (Gitzo G2220/GH2750QR) directly above the center of the plastic container. The direction of the non-blackened side was arbitrarily coded as ‘N’ and the orientation of the beetles’ movement expressed as octants of the cardinal directions (i.e., N, NE, E, SE etc.). The movement of the beetles was scored per octant when the beetle reached the edge of the paper (and essentially the bounding wall of the chamber) or until the end of recording.

For experiment #1 the average crawl speed of the beetles was ascertained in mm/s. Using the video time stamps, individual beetles were timed as they traversed a distance of 100 mm between the concentric circles. Ten beetles were recorded, each crawling in a direct line towards either N (light) or S (dark). Experiment #2 measured the crawl speed of all beetles that moved towards one of the colored fields. To avoid a situation where other beetles might influence the crawl directions and speeds of the recorded beetles, time recording (i) only commenced once a beetle had passed the ‘start line’ set at the 25 mm concentric circle and (ii) involved only those beetles that moved without coming in contact with other beetles after they had passed the start line.

### 2.4. Illumination Measurements

Illumination was assessed retrospectively using a PocketLab Traveller probe. The luminosity of the of the various surfaces (except ambient light) was measured with the probe at a distance of 50 mm. The luminosity of the two LED lights was measured at distances of 0–150 mm (in 25 mm increments).

### 2.5. Statistics

The significance assessment of observed differences used the Chi-squared test with *n* − 1 correction of the MEDCALC comparison of proportions calculator [42,43,44]. The average crawl speeds were compared with the student’s T-TEST function in MS Excel.

## 3. Results

### 3.1. Experiment #1

**Figure 5:**
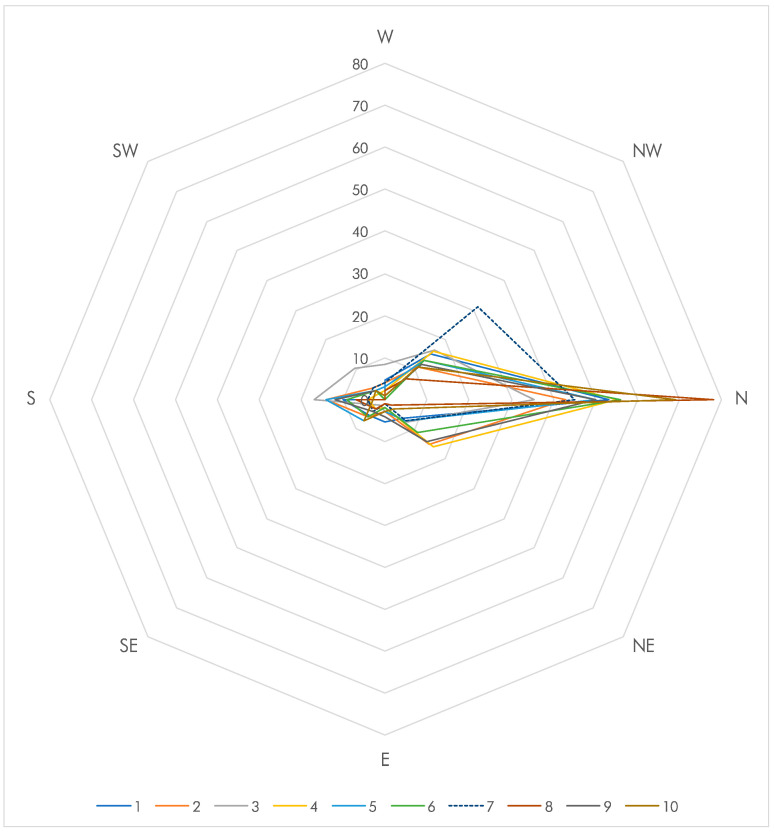
Radar plot showing the final distribution of beetles across the octants (in %). Each color represents the distribution of a replicate (numbered 1–10).

The observed beetle movements in the octants are shown in Table 2 in comparison to a theoretical even distribution (12.5% per octant). Between 36% and 78% of all beetles moved towards the lightest octant (N), with between 60% and 87% significantly preferring the light (NW, N, NE) over the dark octants (SE, S, SW) (*p* < 0.0001 for each replicate). The midpoints (E, W) attracted the least number of beetles (5.7 ± 2.9) albeit not significantly so. When considering the octants individually, the N octant consistently attracted more beetles than any other octant (53.5 ± 12.4%, median 52), which was significant for each replicate (chi square texts, *p* = 0.0001 or better), whereas both midpoints (E, W, 5.7 ± 2.9%, median 5) as well as octant SW (3.2 ± 2.9%, median 3) attracted significantly fewer than expected (Table 2). The overall pattern is very similar between the ten replicates (Figure 5) with the exception of replicate #7, which shows a deviation in the NW octant.

The overall documented average crawl speed was 3.50 ± 0.53 mm/s (range 1.82–4.55; median 3.57; *n* = 200). A comparison of the overall crawl speeds towards the light and dark sectors (10 beetles each) showed that *C. dactyliperda* crawled significantly faster towards the light than the dark (*p* ≤ 0.0001) (Table 3). The faster speeds towards the light are not universal, however, as in two replicates the reverse was true (although not significant), and in another four of the ten replicates the speed differences were not significant (Table 3). 

### 3.2. Experiment #2

The observed beetle movements towards reflective color patches by octant are shown in Table 4. While there are differences between the observed and the theoretical frequencies (12.5% per octant), these differences are, on the whole, not significant. The only significant differences were observed in a red-blue octant (replicate #6) and in a blue-green octant (replicate #9), where the values were significantly below expectations. These can be ignored in the larger picture, however, as these combinations occurred in five additional replicates each without exhibiting any significant differences.

The overall documented average crawl speed was 2.80 ± 0.58 mm/s (range 1.47–4.55; median 2.86; *n* = 357) (Table 5), which is significantly slower (*p* < 0.0001) than the average crawl speed observed in experiment #1 (Table 3). While experiment #2 showed minor differences between the colors (Table 5), these are not significant.

### 3.3. Experiment #3

The experiment used single-point diodes to assess the photosensitivity of *C. dactyliperda*. The observed beetle movements in the octants are shown in Table 6 and Table 7, both in absolute numbers of observations and in terms of the significance of the deviation from a theoretical, even distribution (i.e., 12.5% per octant). The octant with the blue LED attracted significantly more individuals than the theoretical mean in two of the three replicates (#1, #3), as did an adjacent octant in replicate #3 (Table 6). The octant with the blue LED attracted significantly more individuals in only one of the three replicates. The difference between the combined light and the opposing combined dark group of octants (Table 6) is significant for both the blue LED (*t*-test, two-tailed paired, *p* = 0.0335) and the red LED (*p* = 0.0308). The difference between the attractiveness of red LEDs compared to blue LEDs, however, is not significant (*p* = 0.2439).

In the set up with a blue LED and an opposing red LED (Figure 4), the octant with the blue LED attracted significantly more individuals than the sector with the red LED (Table 7). When the adjoining octants are included, however, the numbers in the blue sector are not significantly higher than in the red sector (*p* = 0.0685).

## 4. Discussion

Female *C. dactyliperda* only leave the seed in which they breed (or were born) to colonize fresh seeds [12]. Previously, it had been observed that emergence from seeds occurred statistically significantly more frequently during the hours of the night than of the day [14]. As external factors of temperature or humidity between the artificially controlled day and night could be excluded, it was speculated that nocturnal emergence was an instinctive predator avoidance strategy. Yet, anecdotal observations had shown that once exposed, *C. dactyliperda* were actually drawn to lighter environments.

This is the first study where *C. dactyliperda* were systematically subjected to aspects of photosensitivity. The results of the ten replicates of experiment #1 appear unequivocal: *C. dactyliperda* were not only significantly more likely to be attracted to the direction with the brightest light but they also crawled significantly faster in that direction. Attraction to light could also be confirmed in experiment #3, where the light source was a point LED in total darkness rather than a large diffusely lit area. The black-box experiments with the single red and blue LEDs, as well with the combined LEDs, showed that the beetles were attracted to the light source per se. While blue LEDs exert a greater attraction than red LEDs, as shown in mono-color and bi-color experiments, it is not significantly so. The difference can be explained by the fact that the blue LED had a higher luminosity than the red LED (Figure 3) and hence a greater light cone (Figure 2).

The color experiment (#2) reported here was based on light reflected off colored semi-matte cardboard surfaces. The study showed no preference of one color over another either in the direction or the speed of movement.

This pilot study has shown that *C. dactyliperda*, although a cryptic species that only emerges from the seeds to disperse, while preferentially eschewing emergence from seeds during daylight hours, *are* attracted to light.

The biological and behavioral determinants of why *C. dactyliperda* is attracted to light remain unclear at present. It can be speculated that because of their cryptic life cycle, the species has lost common predator avoidance strategies such as hiding in darker spaces. The observation, however, provides an opportunity to explore control measures by exploiting the beetle’s photosensitivity to trap dispersing *C. dactyliperda* with a combination of light and pheromone traps.

## 5. Conclusions

*Coccotrypes dactyliperda* were found to be attracted to light sources, which was surprising given that they spend almost their entire life cycle as a cryptic species inside palm seeds and given that the location of new seeds for colonization relies on kairomones rather than visual cues.

Future studies should consider the influences of light intensity, using a smaller point source of light (but with a diffused target), which allows both the wavelength and the luminosity of the light source to be modulated as well as assess the influence of reflectance.

## Figures and Tables

**Table 1:** Experiment #2. Sequence of placement of sector colors.

Replicate	Sector 1	Sector 2	Sector 3	Sector 4
1	Blue	Red	Green	Black
2	Red	Green	Blue	Black
3	Black	Blue	Red	Green
4	Green	Blue	Red	Black
5	Red	Black	Blue	Green
6	Black	Green	Red	Blue
7	Red	Green	Black	Blue
8	Blue	Green	Red	Black
9	Green	Black	Red	Blue
10	Red	Black	Blue	Green

**Table 2:** Experiment #1. Movements of beetles to octants.

		Replicate										Theoretical	% of Replicates Significantly	
	Octant	#1	#2	#3	#4	#5	#6	#7	#8	#9	#10	Distribution	Higher	Lower
	NW	15	11	17	16	13	13	31 + +	7	12	11	12.5	10	—
light	N	53 + + +	43 + + +	36 + + +	55 + + +	50 + + +	56 + + +	45 + + +	78 + + +	50 + + +	69 + + +	12.5	100	—
	NE	6	15	7	16	7	11	7	2	14	3	12.5	—	—
midpoint	E	5	3	3 −	2 − −	3 −	2 − −	1 −	1 −	4 −	3 −	12.5	—	80
	W	5	4 −	8	2 − −	3 −	0 − − −	4	2 − −	1 −	1 −	12.5	—	80
	SE	5	6	2 − −	3 −	7	6	4 −	3 −	4 −	7	12.5	—	50
dark	S	10	14	17	4 −	14	9	4 −	7	12	3 −	12.5	—	30
	SW	0 − − −	4 −	10	2 − −	3 −	3 −	4 −	0 − − −	3 −	3 −	12.5	—	90
	Total	100	100	100	100	100	100	100	100	100	100	100		

Significance (*p* value, *χ*^2^ test) of deviation from expectation (all df = 1) coded as −: *p* ≤ 0.05, − −: *p* ≤ 0.01, − − − or +++: *p* ≤ 0.001. Plus signs represent overrepresentation; minus signs indicate underrepresentation.

**Table 3:** Experiment #1. Crawl speeds of *Coccotrypes dactyliperda* (in mm/s) towards light and dark sectors (*p* value based on two-tailed paired *t*-test, significant value show in italics).

	Movement to Light Sector (N)				Movement to Dark Sector (S)				Faster	
Repeat	Min	Avg ± StdDev	Max	*n*	Min	Avg ± StdDev	Max	*n*	Speed	*p*=
1	2.56	3.48 ± 0.55	4.17	10	2.86	3.70 ± 0.44	4.17	10	Dark	0.2147
2	2.78	3.76 ± 0.43	4.17	10	2.86	3.10 ± 0.18	3.33	10	Light	*0.0026*
3	2.17	3.42 ± 0.55	4.00	10	2.38	3.24 ± 0.59	4.00	10	Light	0.5253
4	2.63	3.34 ± 0.46	4.00	10	2.94	3.57 ± 0.51	4.55	10	Dark	0.2877
5	2.94	3.55 ± 0.35	4.17	10	2.56	3.32 ± 0.44	4.00	10	Light	0.0927
6	2.78	4.02 ± 0.62	4.55	10	2.94	3.39 ± 0.44	4.17	10	Light	*0.0184*
7	2.86	3.60 ± 0.47	4.35	10	1.82	3.24 ± 0.70	4.17	10	Light	0.1531
8	3.13	3.83 ± 0.35	4.35	10	2.86	3.53 ± 0.39	4.17	10	Light	0.1131
9	2.86	3.77 ± 0.42	4.17	10	2.86	3.31 ± 0.35	3.70	10	Light	*0.0163*
10	2.94	3.74 ± 0.41	4.35	10	2.22	2.99 ± 0.70	4.17	10	Light	*0.0036*
Total	2.17	3.65 ± 0.49	4.55	100	1.82	3.34 ± 0.52	4.55	100	Light	*0.0000*

**Table 4:** Experiment #2. Movements of beetles to octants.

Color	Rep #1	Rep #2	Rep #3	Rep #4	Rep #5	Rep #6	Rep #7	Rep #8	Rep #9	Rep #10
Red	11	11	10	16	14	17	11	22	17	6
Green	8	11	14	16	8	14	13	15	6	9
Blue	10	14	12	7	18	11	16	10	7	12
Black	11	14	12	14	14	11	13	16	18	18
Red-Green	15	12	17	—	8	13	16	10	—	10
Red-Blue	23	—	16	19	—	3 *	5	—	14	—
Red-Black	—	15	—	8	18	18	—	14	19	15
Blue-Green	—	9	—	8	10	—	—	5	4 *	11
Blue-Black	14	15	11	—	10	15	8	9	14	18
Green-Black	8	—	8	12	—	—	18	—	—	—
*n*	100	100	100	100	100	100	100	100	100	100

Significance (*p* value, *χ*^2^ test) of deviation from expectation (all df = 1) coded as * *p* ≤ 0.05.

**Table 5:** Experiment #2. Crawl speeds of *Coccotrypes dactyliperda* (in mm/s) towards colored sectors.

Color	Min	Avg ± 1*σ*	Median	Max	*n*
Black	1.61	2.82 ± 0.64	2.86	4.55	108
Blue	1.47	2.79 ± 0.60	2.86	4.17	80
Green	1.52	2.76 ± 0.52	2.86	3.70	73
Red	1.56	2.82 ± 0.54	2.82	4.00	96
All	1.47	2.80 ± 0.58	2.86	4.55	357

**Table 6:** Movements of beetles to octants. Experiment #3. Mono color blue. Coding as –: *p* ≤ 0.05, − − or ++: *p* ≤ 0.01, − − − or +++: *p* ≤ 0.001. Plus signs represent overrepresentation; minus signs indicate underrepresentation.

		Mono Color Blue			Mono Color Red		
	Octant	Rep #1	Rep #2	Rep #3	Rep #1	Rep #2	Rep #3
	NW	15	23	31 + +	17	22	21
light	BLUE	31 + +	22	34 + + +	32 + + +	23	17
	NE	13	21	12	11	8	13
midpoint	E	7	6	6	11	0 − − −	8
	W	6	10	4 −	6	14	16
	SE	8	6	2 − −	5	11	8
dark	S	12	8	6	13	10	8
	SW	7	4 −	6	6	13	8
	*n*	100	100	100	100	100	100

**Table 7:** Movements of beetles to octants. Experiment #3. Coding as −: *p* ≤ 0.05, +++: *p* ≤ 0.001. Plus signs represent overrepresentation; minus signs indicate underrepresentation.

	Observations		
Octant	Rep #1	Rep #2	Rep #3
NW	18	8	10
**BLUE**	**45 + + +**	**38 + + +**	**65 + + +**
NE	4	10	4 −
E	5	8	1 −
SE	6	12	1 −
**RED**	**9**	**11**	**6**
SW	6	9	4 −
W	8	6	10
total	100	100	100
